# International Course on Emerging Viruses in the Amazon Region

**DOI:** 10.3201/eid1504.080367

**Published:** 2009-04

**Authors:** Ana Lisa do Vale Gomes, Cintia Magalhães, Fernando Melo, Rocio Inga, Sandra R. Gadelha

**Affiliations:** Fundação Oswaldo Cruz, Recife, Brazil (A.L. do Vale Gomes); Universidade Federal de Minas Gerais, Belo Horizonte, Brazil (C. Magalhães); Unversidade de São Paulo, São Paulo, Brazil (F. Melo); Universidad Peruana Cayetano Heredia, Lima, Peru (R. Inga); Universidade Estadual de Santa Cruz, Ilhéus, Brazil (S.R. Gadelha)

**Keywords:** emerging viruses, Amazon region, international course, conference summary

The international course Emerging Viruses: Global Approaches and Specificities of the Amazon Region was held in Porto Velho, Rondonia, Brazil, November 17–December 7, 2007. Organized as part of the Amsud-Pasteur research collaboration program (sponsored by Institut Pasteur), the course was held there primarily because Rondonia State is located in the Amazon region, a particular environment in which new viruses could emerge as a consequence of ecologic changes brought about by exploration and other human activities.

Speakers with broad expertise from different regions of the world were featured (full list available at in the Technical Appendix). The class was attended by 26 postgraduate students from 16 institutes and universities from Brazil, Argentina, Peru, Paraguay, and Uruguay. The main objective of the course was to equip a generation of young investigators with up-to-date information concerning emerging viruses and to lay the foundation of a research network that would be able to provide the Amazon region with increased responsiveness to future viral disease challenges. The original format of the course made it possible to include not only the scientific community but also health authorities and the general population through a series of workshops and debates.

The course was theoretical and practical and dealt with ecosystems, vectors and viruses, pathogenesis, and control. During these sessions, the most notable emerging viral diseases were discussed, including their geographic distribution, the effects on public health, the lack of treatment options, and current and potential vaccines. Viral diseases were analyzed from multiple perspectives: the virus itself, its molecular biology and mechanisms of infection, disease epidemiology and transmission, viral genetic diversity and evolution, vectors and reservoirs, and the immunologic response and viral escape mechanisms. Furthermore, for each virus family, the various clinical forms of disease, diagnostic methods, available antiviral drugs, and vaccine development were reviewed and discussed.

The viruses considered the most likely to emerge in the Amazon region were reviewed according to family: *Flaviviridae* (yellow fever, dengue, and hepatitis C viruses), *Bunyaviridae* (hantavirus, and Oropouche, Rift Valley fever, and Crimean-Congo hemorrhagic fever viruses), *Rhabdoviridae* (rabies virus), *Filoviridae* (Ebola virus), *Togaviridae* (chikungunya and Mayaro viruses), *Papillomaviridae* (human papillomavirus), *Hepadnaviridae* (hepatitis B virus), *Orthomyxoviridae* (influenza virus), *Coronaviridae* (severe acute respiratory syndrome coronavirus), and *Retroviridae* (human T-cell lymphotropic virus). Among these viruses, the most prevalent in this region are dengue; yellow fever; Oropouche; Mayaro; and hepatitis B, C, and D viruses; however, the economic, social, and ecologic characteristics of the region could promote the emergence of other viruses. In fact, the Amazon Basin is often regarded as an arbovirus sanctuary where dormant or nonpathogenic viruses can find an optimal context to emerge or reinforce their pathogenic potential.

The meetings were attended by local authorities, university students, healthcare workers (physicians, nurses, clinical laboratory personnel, technicians, cleaning and maintenance personnel), and the general population. During this interdisciplinary event, scientists, local authorities, and the general population had the opportunity to share information and exchange experiences, reinforcing the importance of educational programs that are fundamental to control and to identify emerging viral diseases. In fact, the efforts planned and conducted by local authorities and the general population in conjunction with scientists are an essential and indispensable part of public health efforts. For that reason, the creation of a network that was both scientific and para-scientific was a main purpose of this course, to allow all groups to participate in improving the awareness of emerging viruses in the Amazon region.

The course provided a unique opportunity for South American students to confront different views on emerging viruses and to interact with other young scientists from South America. This contact made it possible to establish international and national collaboration. Also, the course sparked new ideas that arose during the meetings and also after the course, as discussions continued while students worked on their PhD projects.

## Supplementary Material

Technical Appendix AMSUD-Pasteur course on: EMERGING VIRUSES : global approaches and specificities of the Amazon region

