# Supplementary material for: International Course on Emerging Viruses in the Amazon Region
Source: Emerg Infect Dis. 2009 Apr;15(4):e1. doi: 10.3201/eid1504.080367 (PMC2671423; doi:10.3201/eid1504.080367)
Supplement: Technical Appendix — AMSUD-Pasteur course on: EMERGING VIRUSES : global approaches and specificities of the Amazon region [file 08-0367_Techapp-s1.pdf]

INSTITUT PASTEUR

AMSUD-Pasteur course on:

EMERGING VIRUSES :
global approaches and specificities of the Amazon region

CHARACTERISTICS of the COURSE:

International course on viral diseases and prevention strategies with a special focus on the Amazon Region in South America. Students are expected from Research Institutes and Universities from South America , specially from the countries of the Amazon area
Conferences and practicals
Students will be admitted on the basis of scientific excellence, academic track record and motivation. Fellowships for travel or travel and housing will be available upon submission of a special application.

LEVEL of the COURSE:
Advanced level: Master/MPhil, PhD, Post-doc in Molecular Medicine (ID), Microbiology, Medicinal Chemistry or Biochemistry

ORGANIZERS

Name and Title of Applicant: **Noel Tordo**, Ph.D.
Address, telephone number and e- mail: Unit Antiviral Strategies, Department of Virology
Institut Pasteur, 25 rue du Dr Roux, 75724 Paris, Cedex 15
ntordo@pasteur.fr Tel: +33140613134 fax:40613256

Name and Title of Co- Applicant: **Jean Louis Virelizier** , MD, Pr
Address, telephone number and e-mail Institut Pasteur, 25 rue du Dr Roux, 75724 Paris Cedex 15
virelizi@pasteur.fr tel +33 1 4568 82 62 fax +33 14568 8941

Name ad Title of Co-applicant **Luiz Hildebrando Pereira da Silva** , M.D.
Instituto de Pesquisa em Patologias Tropicais (IPEPATRO)
Rua da Beira 7671 78970-000 Porto Velho, RO Brazil
hildebrando@cepem.com.br tel/fax 00 55 69 32196012

Name and Title of Co-applicant **Roberto Sena Rocha, M.D.**
Address, telephone and e-mail Centro de Pesquisa Leonidas e Maria Deane, FIOCRUZ
Rua Terezina 476, Adrianopolis, Manaus, 69 057 070, AM, Brazil
srocha@amazonia.fiocruz.br tel 55 92 3621 2390

Emerging viruses. Global approaches and specifities of the Amazonia region
CEPEM, Porto Velho, Brasil
November/December 2007

Domain of the call : Virology / Tropical medicine

Organizers

- Luiz Hildebrando PEREIRA da SILVA (Porto Velho, Brasil)
- Roberta SENA ROCHA (Fiocruz, Manaus)
- Noël TORDO (Institut Pasteur, Paris-Lyon)
- Jean-Louis VIRELIZIER (Institut Pasteur , Paris)

Invited speakers

Marilene F. de ALMEIDA (Sâo Paulo)
Juan ARBIZA (Montevideo) jarbiza@fcien.edu.uy
Albino BELOTTO (PAHO, Washington)
Esteban DOMINGO (Madrid)
Edson DURIGON (USP, Sâo Paulo)
Pierre DUSSARD (Pasteur, Cayenne)
Delia A ENRIA (Pergamino, Argentina) deliaenria@gmail.com
Ana Bella FAILLOUX (Pasteur, Paris)
Silvana R. FAVORETTO (I. Pasteur and USP, Sâo Paulo)
Ricardo GALLER (Fiocruz, Rio de Janeiro) rgaller@ioc.fiocruz.br
Antoine GESSAIN (Pasteur, Paris)
Bernardo GALVAO de CASTRO (Fiocruz, Salvador)
Douglas GOLENBOCK (Mass University, Boston) douglas.gollenbock@umassmed.edu
Eva HARRIS (Berkeley) eharris@socrates.berkeley.edu
Stephen HIGGS (UTMB) sthiggs@utmb.edu
Ricardo ISHAK, R , (UFPa Belem) rishak@ufpa.br
Alan KAY (INSERM, Lyon)
Vincent LACOSTE (Pasteur, Cayenne)
Alexandre C.LINHARES (IEC, Belem) alexandrelinhares@iec.pa.gov.br
Jean-Claude MANUGUERRA (Pasteur, Paris) jmanug@pasteur.fr
Philippe MARIANNEAU (Pasteur, Lyon) marianneau@cervi-lyon.inserm.fr
Rita MEDEIROS (IEC, Belem) ritasousa@iec.pa.gov.br
Veronika von MESSLING (Armand Frappier, Montreal) veronika.vonmessling@iaf.inrs.ca
Marisa G. MORGADO (Fiocruz, Rio, Brazil) – mmorgado@ioc.fiocruz.br
Jacques MORVAN (Pasteur, Cayenne)
F C MOTTA (Fiocruz, Rio de J.) fcmotta@ioc.fiocruz.br
G. PARANHOS-BACCALA (Biomerieux, Lyon)
Nathalie PERDIGON (Pasteur, Paris) perdigon@pasteur.fr
Mitermeyer G. REIS M.G.,(Fiocruz, Salvador) miter@cpqgm.fiocruz.br
Pierre ROLLIN (CDC, Atlanta)
Y. ROTIVEL (Pasteur – Paris)
Debora SACRAMENTO (USP, Sâo Paulo)
Jean F SALLUZZO (Sanofi Pasteur, Lyon).
Claudia N.dos SANTOS (IBPM, Curitiba) clsantos@tecpar.br
Wilson SAVINO (Fiocruz, Rio de Janeiro) savino@fiocruz.br
Hermann SCHATZMAYR (Fiocruz, Rio de J.)- hermann@ioc.fiocruz.br
Amilcar TANURI (URFJ, Rio de Janeiro) atanuri@biologia.ufrj.br
De THOISY (Pasteur, Cayenne)
A C VALLINOTO (UFPa, Belem) vallinoto@ufpa.br
Pedro F VASCONCELOS (IEC, Belem, Para) pedrovasconcelos@iec.pa.gov.br
Ana Paulo C.VICENTE (Fiocruz, Rio, Brazil) anapaulo@fiocruz.br
Luiza L.VILLA (I.Ludwig I., Sao Paulo) – llvilla@ludwig.org.br
Paolo ZANOTTO (USP, Sâo Paulo) pzanotto@usp

Hervé ZELLER (INSERM, Lyon). zeller@cervi-lyon.inserm.fr

INTEREST OF THE COURSE FOR THE REGION:

The Amazon Region is characterized by multiple, original ecosystems including the world largest tropical rain forest with a variety of local specific fauna and flora with a large and rich repertoire of new world species. The rain forest area is being rapidly modified and submitted to increase devastation by the relatively recent introduction of new human populations. Migrating populations, with their domestic animals (cattle in particular) occupy progressively areas where humans were formerly absent or represented only by low density original Amerindian ethnias that had low impact on the natural environment. Such evolving ecosystems facilitate the contact of a variety of microorganisms with new plant and animal hosts including Man, and favor the appearance of new pathologies, in particular virus agents and development of arthropod vectors with emergence or re emergence of new or known diseases.

The Amazon Region is a huge geographical area, with more than 5 millions square kilometers, including, in addition to large areas of Brazil, areas of Paraguay, Bolivia, Peru, Ecuador, Colombia, Venezuela, Guiana, Surinam and French Guyane. In consequence, public health problems associated to periodic emergence of virus infections (Arbo and Retrovirus in particular) as well as dissemination of known infectious agents (HIV, influenza, herpes) affect and concerns all of these countries. Only a transnational program of research and globalized public health actions can efficiently grasp such complex microbiological challenge in such a huge and varied environment.

The present project of an integrated course of tropical virology is , in agreement with the general aim of the AMSUD-PASTEUR research program, to raise a generation of young investigators from that region with modern knowledge in Virology both theoretical and practical, to provide the concerned countries with a research network able to provide the region with a better responsiveness to future virological challenges in the Amazon Region.

The initiative of the course was taken by three scientists from the Pasteur Institute, who will constitute the organizing Committee: Noel Tordo, head of the P4 laboratory (Pasteur, Lyon) , Jean Louis Virelizier, (department of Virology, honorary professor at Institut Pasteur), and Luiz H. Pereira da Silva, formerly head of the Unité de Parasitologie Expérimentale (Pasteur, Paris) , now Director of the Instituto de Pesquisa em Patologias Tropicais (IPEPATRO) in Rondonia, Brazilian Amazon Region. The course is proposed to be held in Porto Velho, capital of the Rondônia State, from November 17th 2007 to December 7th 2007. The responsibility for infra structure and local arrangements will be taken by IPEPATRO and the Amazon branch of FIOCRUZ (Instituto de Pesquisa Leonidas and Maria Deane), through their respective Scientific Directors, namely Roberto Sena Rocha and Rodrigo Guerino Stabeli. The specialized Departments of the Federal Universities of Para, Amazonas and Rondonia States and Rondonia Health State Department (SESAU) have given their agreement for scientific and technical collaboration.

The Organizing Committee proposes to select 24 graduate and post-graduate students from the AmSud region, primarily on the basis of scientific excellence, academic track record and motivation. About half of them will come from various Scientific or Academic Brazilian Institutions, with a particular attention for Institutions of the Amazon Region; the second half will come from other South American Institutions.

Financial support asked to AMSUD Pasteur will be complemented by support of Brazilian Official and Private Agencies and Foundations. Secretary of Health Surveillance , the Department of Science and Technology of the Ministry of Health , and Ministry of Scieces & Technology. Other Institutions will be contacted as soon as the AMSUD PASTEUR agreement will be known.

A further benefit from the course will be the availability of fellowships for Brazilian students , provided by the Brazilian Official Agency CAPES (Ministry of Education), will permit research stays in foreign Virology laboratories, after the Course, either for short stages or for complement doctoral stays (sandwich fellowships) or post-doctoral stays. These measures will be of interest for the development of international collaboration in the area of emergent virus diseases in the Amazon Region

DESCRIPTION OF THE COURSE

DURATION: Three weeks
TYPE (level, theory, practical…): M Sc and Ph.D, Post-doc, theory and practical

LOCATION: Instituto de Pesquisa em Patologias Tropicais (IPEPATRO), Universidade Federal de Rondonia (UNIR), Secretaria de Saúde de Rondonia (SESAU) Porto Velho, RO, Brasil
DATES: 17/11/2007 – 08/12/2007

NUMBER OF STUDENTS: 24
LANGUAGE: English
AVAILABLE EQUIPMENT IN HOST INSTITUTION AND INFRASTRUCTURE
Seminar room:
Video, overhead projectors, access to larger seminar rooms
Special room for student's bibliography search and internet access
12 computers and one printing
Teaching Laboratory:
P1 molecular biology and biochemistry laboratory with *ad hoc* equipment for molecular biology, protein biochemistry, immuno fluorescence microscopy, ELISA equipment …
BSL2 laboratory with 4 cell culture hoods

Preliminary Programme

18 NOVEMBER / 7 DECEMBER 2007, PORTO VELHO, BRAZIL

Week 1

ECOSYSTEMS, VECTORS and VIRUSES

- **Sunday 18 November 2007.**

Registration, gathering and wellcome party

- **Monday 19 Nov.**

*** Morning**
8:00 - 8:30 : Introductory remarks by organizers

8:30 - 10:00 : Esteban DOMINGO (Madrid) : Virus evolution and diversification
10:30 -11:30 : Paolo ZANOTTO (USP, Sao Paulo) : Phylogeny of viruses
12:00 – 12:30 : General discussion

***Afternoon**
14:00 – 15:30 : Juan ARBIZA (Montevideo) : Coordinating Virus Watch in South America
16:00 – 17:00 : Jean-Claude MANUGUERRA (Pasteur, Paris) : Alert and responsiveness to emerging virus infections.
17:00-17:30 : General discussion

18:00 – 19:00 Preparation for thematic seminars : presentation of scientific themes by tutors, expositions of publications (pdf).

Virelizier J-L (Pasteur,Paris) ; Rodrigo G: Stabeli(IPEPATRO, Porto-Velho), N.Tordo (Pasteur, Lyon) , Claudia Dos Santos (IBCP, Curitiba) Rita Medeiros (IEC, Belem), Edmilson Rui (IPEPATRO, PortoVelho)

- **Tuesday 20 nov**

*** Morning**
- 8:00 – 9:30 : Stephen HIGGS (UTMB) : Mosquito/virus/vertebrate interactions
- 10:00 – 11:00 : Anna Bella FAILLOUX (Pasteur, Paris) : Mosquitos as hosts for viruses
- 11:30 - 12:30- Philippe DUSSART (Pasteur, Cayenne) : Flaviviruses circulating in Guyana

*** Afternoon**
14:30 - 15:30: Geographic information System for arbovirus : Jacques MORVAN (Pasteur, Cayenne)
16:00 – 17:00- De THOISY (Pasteur, Cayenne) : Wild terrestrial rainforest mammals as potential reservoirs for flavivirus
17:00- 17:30 General discussion

18:00 – 18:30 **Student group definition, description of thematic seminars**

- **Wedn 21 nov**

*** Morning**
- 8:30 – 10:00 – Pedro VASCONCELOS (IEC, Belem) : Flaviviruses in the Amazonian delta
- 10:30 – 12:00 – Nathalie PERDIGON (Pasteur, Paris) Pathogenesis of flavivirus infections

*** Afternoon**
- 14:00 –15:30 – Ricardo GALLER (Fiocruz, Rio de Janeiro) : new vaccines against yellow fever and West Nile virus
- *15:30-16:30 General discussion*- Chair : H. SCHATZMAYR (Fiocruz, Rio de Janeiro)
- 17:00-18:30: A. KAY (Inserm, Lyon): Hepatitis viruses: specific focus on the Amazonas Region.

- **Thurs 22 Nov**

*** Morning**
- 8:30 – 9:30: G. PARANHOS-BACCALA (Biomerieux, Lyon) Diagnosis of Hepatitis viruses in humans using serological, virological and genetic methods.
- *9:30 - 10:00 General discussion- Chair : G. PARANHOS BACCALA (Biomerieux, Lyon -)*
- 10:30-12:00- Vincent LACOSTE (Pasteur, Cayenne) : Novel herpes viruses in non human primates

*** Afternoon**
14:00-15:30- GALVAO CASTRO , B (Fiocruz, Salvador): HTLV-1 in Brazil
16:00-17:30- ISHAK, R , (UFPa, Belem,) : HTLV-2 in Brazil
17:30-18:30 General discussion

- **Friday 23 Nov**

*** Morning**
8:30 – 10:00 - Noël TORDO (Pasteur, Paris-Lyon) : Carnivores, bats and Lyssaviruses
10:30 – 11:30 : Albino BELOTTO (PAHO, Washington) Towards elimination of dog rabies in the Americas : a constant politicaleffort supported by PAHO.
11:30-12:30 : Debora SACRAMENTO (USP, Sao Paulo) : Molecular epidemiology of rabies virus in human, domestic animals and wildlife in Brazil.

*** Afternoon**

14:00-15:00 – Wilson UIEDA (UNESP, São Paulo). The bat fauna and distribution of vampire bats in amazonian region
15:00-16:00 – Marilene FERNANDES de ALMEIDA, (Sâo-Paulo) : Desmodus rotundus: biology, behaviour and alternative for population control.
16:30-17:30 –: R. MEDEIROS (IEC, Belem): Bat-transmitted human rabies in Brazilian Amazon.
17h30-18h30 General discussion (Discussant : - Y. ROTIVEL (Pasteur - Paris)

- **Saturday 24 Nov**

*** Morning :**
10h-12h00 *:* **: 1st Work Meeting between student groups and Tutors.**

Afternoon : free

- **<u>Sunday 25 Nov</u>** : free

Week 2

- **<u>Monday 26 Nov</u>**

*** Morning**
- 8:30 – 10:00 -. Pierre ROLLIN (CDC, Atlanta) : Ecosystems and virus emergences
10:30 – 12:00 – Hervé Zeller (CCOMS, Lyon) : Japanese Encephalitis Virus as a model of emerging arbovirus infection
12:00-13:00 General discussion : Secure handling of emerging pathogens in the wild and in the lab.

*** Afternoon**

Practicals, day 1 : Pierre ROLLIN (CDC, Atlanta) + Philippe MARIANNEAU (Pasteur, Lyon)
Comparing serology, antigen detection and molecular methods in the diagnostic and characterisation of pathogenic viruses

PERSISTENT INFECTIONS

- **<u>Tuesday 27 Nov</u>**

*** Morning**
8:00-9:30 - J-L VIRELIZIER (Pasteur, Paris) HIV strategy of persistence and dissemination
10:00-11:00- M.G. MORGADO (Fiocruz, Rio de Janeiro) : HIV-1 subtype dissemination in Brazil.
11:00-12:00- Amilcar TANURI (UFRJ, Rio de Janeiro) : HIV drugs and resistance
12h00-13h00 : General discussion

*** Afternoon**
Practicals, day 2

- **<u>Wedn 28 Nov</u>**

*** Morning**
8:30-10:00- Luiza L VILLA (Ludwig I., Sao Paulo) : Chronic HPV infection and its prevention by vaccines
10:30-12:00- Mitermeyer REIS (Fiocruz, Salvador) : HCV prevalence and genotypes in Northern Brazil

12:00- 13:00: General discussion

*** Afternoon**
Practicals, day 3

- **Thursday, 29 Nov**

Host and virus genetics : impact on pathogenesis

8:30-10:00 -VALLINOTO A.C. (UFPa, Belem) **:** Coinfections with retroviruses in the genetic context of Amazonian indians.
10:30-11:30 - VICENTE A.C. (Fiocruz, Rio de Janeiro) : Proliferative advantage of the « Brazilian » HTLV-1 p12 virus signature
General discussion

*** Afternoon**
Practicals, day 4

- **Friday 30 nov**

8:30-10:00 : Antoine GESSAIN (Pasteur, Paris) : Tropical forest and virus emergences

*** Morning + afternoon**
Practicals, day 5

*** Afternoon**
16:00-18:00 : **2nd Work Meeting between student groups and Tutors.**

- **Saturday , 1 December; free**
- **Sunday, 2 December : free**

WEEK 3

ACUTE , PATHOGENIC INFECTIONS AND THEIR CONTROL

- **Monday , 3 December**

*** Morning**
8:00-9:30 - Sylvie VAN DER WERF (Pasteur, Paris) : Influenza virus : replication and pathogenicity
10:00-11:00 - Veronika von MESSLING (Armand Frappier, Montreal) The ferret model for investigating the pathogenicity of influenza viruses.
11:30-12:30 - Rita MEDEIROS (IEC, Belem) : Host range restriction of the influenza haemagglutinin.

*** Afternoon**
14:00-15:00 : MOTTA F.C. (Fiocruz, Rio de Janeiro) . Circulation of influenza A,B and C in Brazil.
15:30-17:00 : JF SALLUZZO (Sanofi Pasteur, Lyon). Vaccines against influenza viruses
17:00-18:00 : General discussion :Preparadness against pandemics : prevention measures, vaccines and antivirals

- **Tuesday 4 December**

*** Morning**
8:30-10:00- LINHARES A C (IEC, Belem) : Vaccine against rotavirus

10:30-11:30- ENRIA D. (Pergamino, Argentina) : Identifying hantavirus in rodent reservoirs and humans
11:30-12:30-dos SANTOS C N (IBPM, Curitiba) : Phylogenic analysis of hantaviruses in South America

*** Afternoon**
14:30-16,00 : Edson DURIGON (USP, Sao-Paulo) : Viral ecology in Brazil.
16:00-18:00 : General discussion

- **Wednesday 5 December**

*** Morning**
8:30-10:00-: D. GOLENBOCK (Mass University, Boston) : innate immunity and infections
10:30-12:00- Wilson SAVINO (Fiocruz, Rio-de-Janeiro): Thymus and viruses
12:00-13:00 : General discussion (Discussant : JL VIRELIZIER (Pasteur, Paris)

* Afternoon :
Preparation to seminar presentation (3rd Work Meeting between student groups and Tutors) and oral exam

- **Thursday 6 December**

*** Morning**
Seminar 1
Seminar 2
*** Afternoon**
Seminar 3
Seminar 4

- **Friday 7 December**

*** Morning**
Examination of students
*** Afternoon**
Examination of students

End of the course

LODGING FACILITIES FOR TEACHERS AND STUDENTS

Student will be housed in hotels reserved through IPEPATRO at a special rates. Teachers will be housed in hotels. Three nights are counted for a teacher who gives a lecture. Other periods will be allowed for collaborators in practical courses and seminar preparation.

Send application forms to :
Rodrigo Guerino Stabeli, Scientific Director
Instituto de Pesquisa em Patologias Tropicais (IPEPATRO)
Rua de Beira 7671, Km 3.5, Porto Velho, 78970 000 RO, BRASIL

TEL/Fax: 55 (69) 3219 6010
E-mail: stabeli@iapepatro.org.br or stabeli@unir.br
